# Transcriptome Profiling and Identification of the Candidate Genes Involved in Early Ripening in *Ziziphus Jujuba*

**DOI:** 10.3389/fgene.2022.863746

**Published:** 2022-06-14

**Authors:** Baiyun Li, Hui Li, Zehua Xu, Xinnian Guo, Tao Zhou, Jiangli Shi

**Affiliations:** ^1^ Horticulture Institute, Ningxia Academy of Agricultural and Forestry Sciences, Yinchuan, China; ^2^ Institute of Agricultural Resources and Environment, Ningxia Academy of Agricultural and Forestry Sciences, Yinchuan, China; ^3^ College of Horticulture, Henan Agricultural University, Zhengzhou, China

**Keywords:** jujube, early ripening, transcriptome analysis, transcription factor, differential expression genes

## Abstract

The early ripening jujube is an immensely popular fresh fruit due to its high commercial value as well as rich nutrition. However, little is known about the mechanism of jujube fruit’s ripening. In this study, the transcriptome profiles were comprehensively analyzed between the ‘Lingwu Changzao’ jujube and its early-ripening mutant during the fruit development and maturity. A total of 5,376 and 762 differentially expressed genes (DEGs) were presented at 80 and 90 days after the flowering of the jujube fruit, respectively. Furthermore, 521 common DEGs were identified as candidate genes that might be associated with the fruit’s early ripening. Our findings demonstrated that in a non-climacteric jujube fruit, abscisic acid (ABA) was more greatly involved in fruit ripening than ethylene. Meanwhile, the fruit ripening of the early-ripening mutant was regulated by eight promotors of DEGs related to glucose and fructose, seven repressors of DEGs related to brassinosteroid signal transduction, and a series of transcription factor genes (MYB, Bhlh, and ERF). Additionally, the expression of 20 candidate DEGs was further validated by real-time PCR during the late fruit maturation stage. Collectively, the present study sheds light on the metabolic mechanism of the fruit’s early ripening and provides valuable candidate genes for the early-ripening mutant’s breeding.

## Introduction

Jujube (*Ziziphus jujuba* Mill.) belongs to the *Rhamnaceae* family. China, one of the world’s most important origin centers of *Z. jujuba*, has a long history of cultivation and diverse germplasm resources, and the jujube fruit is commonly utilized as a food and food additive due to its high nutritional value (Gao et al., 2013). The high content of vitamin C in the jujube fruit has greatly attracted people’s interest in view of an important vitamin source for human nutrition. Moreover, dried jujube fruits have been used in traditional Chinese medicines for over 4,000 years (Pareek, 2013; Huang et al., 2016; Dong et al., 2022).

Among fresh fruit crops, the early-ripening cultivars generally have stronger market competitiveness; thus, early ripening is one of the important breeding goals. Fruit ripening includes a series of physiological and biochemical processes. Growing reports suggested that plant hormones, such as jasmonic acid, ABA, and ethylene, are closely linked with fruit development and ripening (Zhang et al., 2009; Jia et al., 2016; Kou et al., 2021). Nowadays, transcriptomic analysis has also been used to explore the early ripening of fruit crops. Nawaz et al. (2021) revealed that the DEGs of 13 plant hormones and 26 transcription factors were responsible for an apple’s early ripening. Xi et al. (2017) reported that the higher overall oxidation status in the grape cultivar ‘Fengzao’ (the early-ripening bud mutant from ‘Kyoho’) could promote the early ripening of the ‘Kyoho’ berry. Terol et al. (2019) also found that a MADS-box transcription factor was involved in the regulation of early ripening in mandarins. Additionally, Ghayoumi et al. (2020) found that the genes SEP1 and NCED6 improved the early ripening of the ‘Yaghooti’ grape. However, the molecular evidence for the early ripening of *Z. jujuba* is lacking.

In recent years, increasing advances in transcriptome and metabolome have been used to explore the function of gene family members in *Z. jujuba* Mill, such as the MYB superfamily (Qing et al., 2019), AP2-ERF superfamily (Zhang and Li, 2018), bZIP family (Zhang et al., 2020), and SPL family (Shao et al., 2017). However, the transcription factors’ family in plants contain a large number of members and participate in a complex regulatory network as an important component. To date, no reports have focused on jujube ripening using the transcriptome method. ‘Lingwu Changzao’ (LC) is a fresh jujube cultivar, originating from the Ningxia Province of China. It is highly popular among consumers for its higher edible rate and rich nutrition and has become a pillar of the local and new agriculture industry development in Ningxia Province (Chen and Cao, 2018). In 2015, our research group found an early-ripening mutant from LC trees, showing half-red fruit in late August, and full-red fruit in early September, while the LC fruit was at a white mature stage. Based on our consecutive investigation in recent years, the early-ripening mutant fruits ripened 20 or so days earlier than LC. Therefore, the current study focused on the comparative transcriptome analysis from ‘Lingwu Changzao’ and its early-ripening mutant to screen the key genes modulating the early ripening of the jujube fruit, which would provide new insights into the early ripening of fruit crops.

## Materials and Methods

### Plant Materials

**FIGURE 1:**
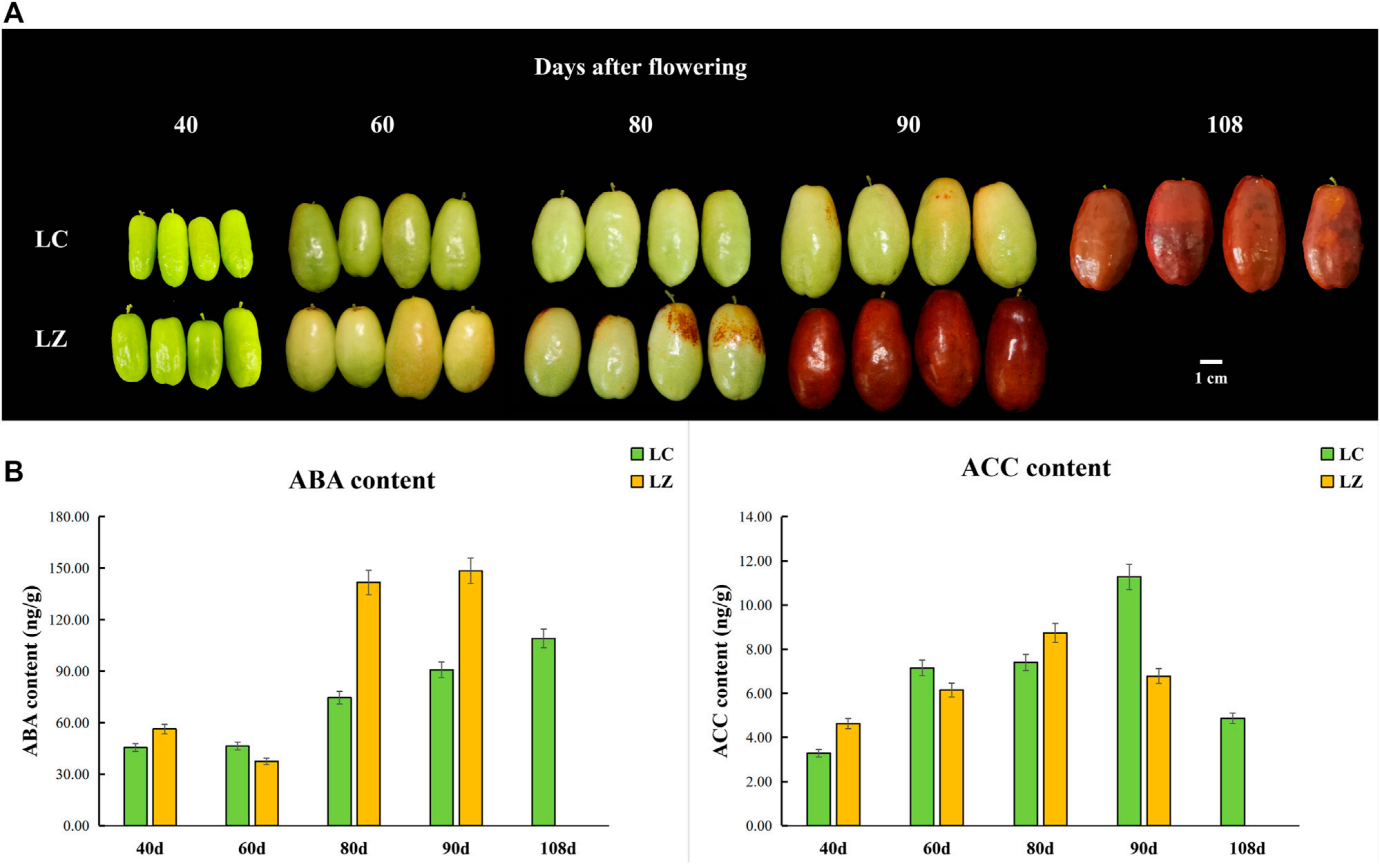
Morphological characteristics and phytohormone trend at different fruit development stages (days after flowering) from the ‘Lingwu Changzao’ jujube (LC) and its early ripening mutant (LZ). **(A)** Morphological characteristics of jujube fruit. **(B)** The contents of ABA and ACC.

‘Lingwu Changzao’ (LC) (*Z. Jujuba* M.) and its early-ripening bud mutant were grown in Lingwu Horticultural Station, Dongta Town, Lingwu City, Ningxia Province, China. No special cultural practices were applied. Lingwu City experiences typically continental monsoon climate. The average annual precipitation is 206.2–255.2 mm, and the annual average temperature is 8.8°C. In 2015, our research group found that the fruit stalk began to be colored from mid-August, the fruit appeared half-red in late August, and fully red in early September, while LC fruit was at the white mature stage. The early-ripening mutant was consecutively investigated from 2016 onward. We found that its fruits ripened 20 or so days earlier than LC fruits, and leaves fell 10 days earlier than LC. In 2018, the marked early-ripening branches were grafted to LC. The early-ripening mutant was named ‘Lingzhou No.1’ (LZ). The fruits from LC and LZ were collected 40, 60, 80, 90, and 108 days after flowering in 2019, respectively, including the enlargement stage, white maturity stage (no red color on the peel), half-red stage, and full-red stage of fruit development (Figure 1A).

### Determination of Fruit Traits

Three biological replicates (five fruit per replicate) were analyzed for the fruit traits. The fruit weight was calculated from 15 fruits with JY-502 electronic balances (Shanghai, China) with an accuracy of ±0.01 g.

The vitamin C (Vc) content was acquired by a 2, 6-dichlorophenol indophenol (DCIP) titration method. Vc content was determined by the consumed volume of the DCIP solution. The titratable acid (TA) was measured using the acid–base neutralization titration method by titrating 5 ml of juice to reach the endpoint of pH 8.2 with 0.1 N NaOH and recording the titration volume. The resulting data were expressed as a citric acid percentage.

The soluble solid content (SSC) was analyzed from the homogenized jujube pulp juice (15 fruits in 100 ml distilled water) using a handheld refractometer (Aita Co., Ltd., Japan). The formula is V/W×A×100% (V means 100 ml, W means fruit weight, and A for the value is from the refractometer).

### Determination of ABA and 1-aminocyclopropane-1-carboxylic Acid (ACC) Contents

The contents of ABA and ACC were investigated by Biomarker Technologies Co., Ltd (Beijing, China). About 50 mg of fresh jujube fruit powder was put into a 2 ml plastic microtube, frozen in liquid nitrogen, and dissolved in 1 ml methanol/water/formic acid (15:4:1, V/V/V). The 10-μL internal standard mixed solution (100 ng/ml) was added to the extract as internal standards (IS) for the quantification. The mixture was vortexed for 10 min and then centrifuged for 5 min at 4°C (12,000 r/min). The supernatant was transferred to clean plastic microtubes, followed by evaporation to dryness, dissolved in 100 μL 80% methanol (V/V), and filtered through a 0.22-μm membrane filter for further LC-MS/MS analysis.

The sample extracts were analyzed using a UPLC-ESI-MS/MS system (UPLC, ExionLC™ AD; MS, Applied Biosystems 6,500 Triple Quadrupole). The analytical conditions were as follows: Waters ACQUITY UPLC HSS T3 C18 (100 mm × 2.1 mm i.d, 1.8 µm); solvent system, water with 0.04% acetic acid (A) and acetonitrile with 0.04% acetic acid (B); gradient program, started at 5% B (0–1 min), increased to 95% B (1–8 min), 95% B (8–9 min), finally ramped back to 5% B (9.1–12 min); 0.35 ml/min flow rate, 40°C, and 2 μL injection volume.

Linear ion trap (LIT) and triple quadrupole (QQQ) scans were acquired on a triple quadrupole–linear ion trap mass spectrometer (QTRAP), QTRAP® 6500+ LC-MS/MS System, equipped with an ESI Turbo ion-spray interface, operating in both positive- and negative-ion mode and controlled by Analyst 1.6.3 software (Sciex). Multiquant 3.0.3 software (Sciex) was used to quantify all metabolites. Mass spectrometer parameters including the declustering potential (DP) and collision energy (CE) for individual MRM transitions were done with further DP and CE optimization. A specific set of MRM transitions was monitored for each period according to the metabolites eluted within this period.

### Library Construction and Transcriptome Sequencing

The total RNA of fruit samples was extracted using a Uniq-10 Column Trizol Total RNA isolation kit (Sangon Biotech Co., Ltd.). RNA concentration and purity were measured using NanoDrop 2000 (Thermo Fisher Scientific, Wilmington, DE). RNA integrity was evaluated using the RNA Nano 6000 assay kit of the Agilent Bioanalyzer 2100 system (Agilent Technologies, CA, United States ). Sequencing libraries were generated according to the procedure of NEBNext UltraTM RNA Library Prep Kit for Illumina (NEB, United States ). In order to select cDNA fragments of preferentially 240 bp in length, the library fragments were purified with the AMPure XP system (Beckman Coulter, Beverly, United States ). USER Enzyme (NEB, United States ) of 3 μL was used with size-selected, adapter-ligated cDNA at 37 °C for 15 min followed by 5 min at 95°C before PCR. Then PCR was performed with Phusion High-Fidelity DNA polymerase, universal PCR primers, and index (X) primer. PCR products were purified (AMPure XP system), and library quality was assessed on the Agilent Bioanalyzer 2100 system.

The clustering of the index-coded samples was performed on a cBot Cluster Generation System using TruSeq PE Cluster Kit v4-cBot-HS (Illumia, United States ) according to the manufacturer’s instructions. After cluster generation, the library preparations were sequenced on an Illumina platform and paired-end reads were generated.

### Transcriptomic Data Analysis

The raw reads were further processed using a bioinformatic pipeline tool, BMKCloud (www.biocloud.net) online platform. Clean data (clean reads) were obtained by removing reads containing adapter, reads containing ploy-N, and low-quality reads from raw data. Meanwhile, Q20, Q30, GC content, and sequence duplication levels of the clean data were calculated. All the downstream analyses were based on clean data with high quality. Only reads with a perfect match or one mismatch were further analyzed and annotated according to the reference genome. Hisat2 tools were used to map with the reference genome.

Gene function was annotated based on Nr (NCBI nonredundant protein sequences), Nt (NCBI nonredundant nucleotide sequences), Pfam (protein family), KOG/COG (Clusters of Orthologous Groups of proteins), Swiss-Prot (A manually annotated and reviewed protein sequence database), KO (KEGG Ortholog database), and GO (Gene Ontology) databases. Gene expression levels were estimated by fragments per kilobase of transcript per million fragments mapped (FPKM). The GO enrichment analysis of DEGs was carried out by GOseq R packages based on Wallenius noncentral hypergeometric distribution. KEGG enrichment was analyzed on the website http://www.genome.jp/kegg/ and the statistical enrichment of DEGs in KEGG pathways was evaluated using KOBAS software.

Differential expression analysis of two samples was conducted using the DESeq2. The resulting *p* values were adjusted using Benjamini and Hochberg’s approach for controlling the false discovery rate. The genes with an adjusted *p*-value <0.05, fold change >2, and FDR <0.01 were assigned as differential expressions. The differential expression analysis of two samples was performed using edgeR software. Significantly differential expression was set as FDR <0.05 and fold change ≥1.5.

### Real-Time PCR (RT-PCR) Validation

Total RNA was extracted as the aforementioned library construction. Total RNA was reverse-transcribed using TUREscript 1st Strand cDNA Synthesis Kit (Imagene). Among the identified DEGs, 20 DEGs were analyzed using the RT-PCR method. The 18S gene (LOC112490218) was used as an internal control. The primer sequences were designed using Primer 6 (Supplementary Table S1). Amplification reactions were performed on a qTOWER2.2 PCR cycler (Analytik Jena, Germany). Cycling parameters were 95°C for 3 min, 39 cycles of 95°C for 10°s, 60°C for 30 s, and 72°C for 30 s. Three biological replicates were performed for each gene.

## Results

### The Fruit Characteristics of LZ and LC

At the commercial mature stage, the fruits were collected from LZ on Sept. 19 and LC on Oct. 6 in 2019. From Table 1, it can be seen that LC fruits had higher single fruit weight and titratable acid, but lower vitamin C content and SSC, than LZ.

**TABLE 1:** Fruit traits’ investigation from the ‘Lingwu Changzao’ jujube (LC) and its early-ripening mutant ‘Lingzhou No.1’ (LZ) in 2019.

Trait	LC	LZ
Single fruit weight (g)	16.09a	15.60a
V content (mg/100g)	217.52b	287.84a
Titratable acid (%)	0.45a	0.38b
SSC (%)	25.10a	26.90a

Fruit ripening and senescence are finely controlled by plant hormones such as ethylene and ABA. As shown in Figure 1B, ABA content increased with fruit development and ripening, and the peaks appeared at the commercial stage of LC and LZ. Moreover, ABA contents in LZ fruits were obviously higher than in LC fruits at 80 and 90 days after flowering. The peak of ACC content was at 10 and 18 days before the ripening for LZ and LC, respectively.

### Illumina Sequencing and Mapping to the Reference Genome

In order to clarify the transcriptional changes in the early-ripening mutant, eight cDNA libraries were constructed, respectively, and the transcriptional difference between LZ and LC was determined by Illumina sequencing. A total of 61.27 GB of clean data were obtained from eight cDNA libraries. Moreover, the clean data of each sample reached over 5.91 GB, and the percentage of Q30 bases exceeded 94.48% (Table 2). The comparison efficiency was 85.77–89.78% in the comparison with the reference genome. After removing the unqualified reads and adapters, 48,325,965; 19,756,582; 21,966,821; 23,231,458; 24,891,589; 21,148,349; 20,081,614; and 25,874,181 high-quality reads were obtained for LC-80d_1, LC-80d_2, LZ-80d_1, LZ-80d_2, LC-90d_1, LC-90d_2, LZ-90d_1, and LZ-90d_2, respectively (Table 2). These results showed that the sequencing quality was sufficiently high for further analysis.

**TABLE 2:** Characteristics of RNA-seq results of different samples.

Sample	Total reads	Mapped reads	Clean reads	Clean bases	GC content (%)	%≥Q30
LC-80ds_1	96,651,93	82,901,362 (85.77%)	48,325,965	14,341,858,050	45.09	95.30
LC-80d_2	39,513,16	35,464,122 (89.75%)	19,756,582	5,909,910,842	44.45	94.67
LZ-80d_1	43,933,64	39,373,277 (89.62%)	21,966,821	6,562,794,378	44.56	94.67
LZ-80d_2	46,462,916	41,535,466 (89.39%)	23,231,458	6,946,238,898	44.25	94.48
LC-90d_1	49,783,178	44,599,816 (89.59%)	24,891,589	7,449,398,784	44.77	94.62
LC-90d_2	42,296,698	37,814,431 (89.40%)	21,148,349	6,324,790,622	44.58	94.72
LZ-90d_1	40,163,228	36,056,862 (89.78%)	20,081,614	6,009,046,582	44.81	94.74
LZ-90d_2	51,748,362	46,395,330 (89.66%)	25,874,181	7,733,070,688	44.51	94.86

Notes: LC-80d and LC-90d represented 80 d after flowering and 90 d after flowering from ‘Lingwu Changzao’ jujube fruits, respectively. LZ-80d and LZ-90d represented 80 d after flowering and 90 d after flowering from the early-ripening mutation ‘Lingzhou No.1’, respectively.

### DEG Identification

**FIGURE 2:**
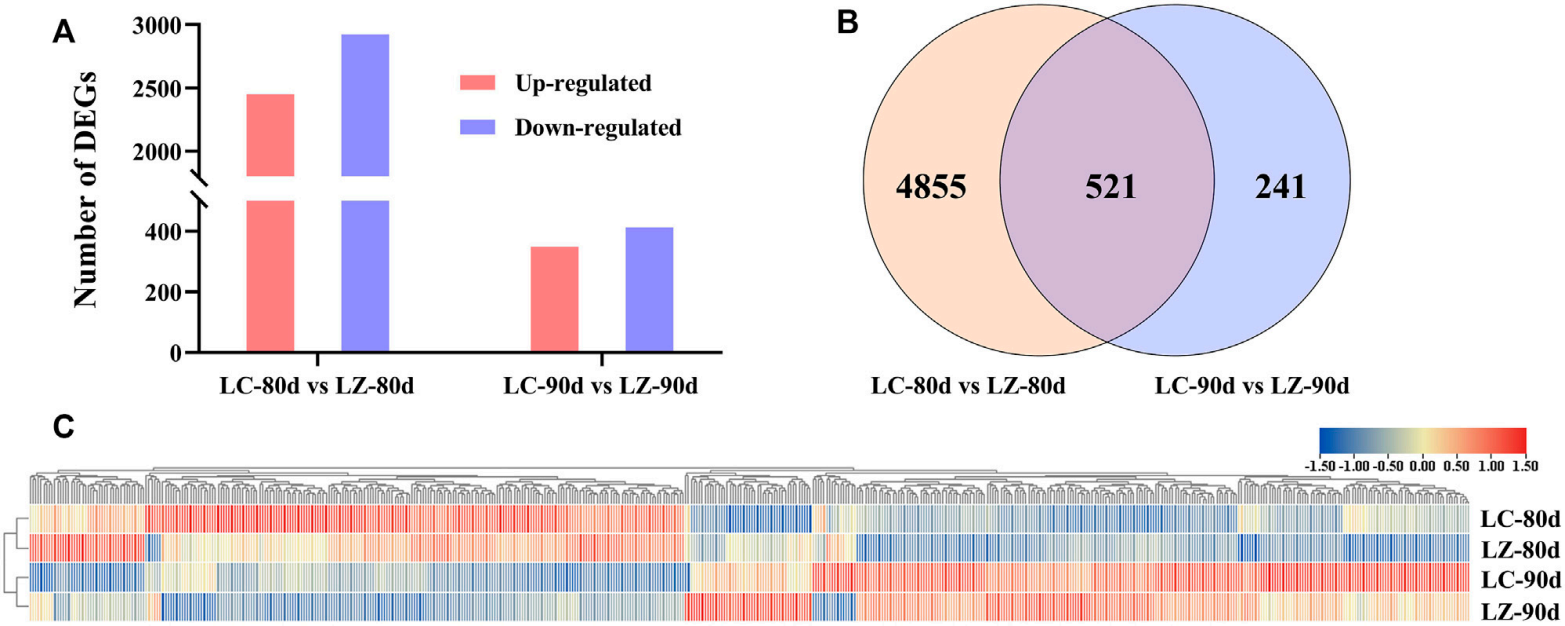
DEG comparison between LC and LZ. **(A)** Numbers of upregulated and downregulated DEGs at 80 days after flowering and 90 days after flowering of the early-ripening mutant LZ. **(B)** Venn diagrams of DEGs at 80 days after flowering and 90 days after flowering of the early-ripening mutant LZ. **(C)** The expression levels of common DEGs between LC and LZ using the hierarchical clustering analysis.

To investigate the differential expression patterns of mRNA in early-ripening jujube fruits, the fruits at 80 and 90 days after flowering were selected because of their obvious difference in fruit skin appearances. The genes with an adjusted *p*-value <0.05, fold change >2, and FDR <0.01 were considered as DEGs. A total of 5,376 DEGs (including 2,450 upregulated DEGs and 2,926 downregulated DEGs) were found in LC-80d vs. LZ-80d, and 762 DEGs (including 349 upregulated genes and 413 downregulated genes) in LC-90d vs. LZ-90d (Figure 2A). From Figure 2B, the Venn diagram exhibited that 521 DEGs were common in the two developmental stages, and 4,855 and 241 DEGs were specifically presented at 80 and 90 days after flowering, respectively. Furthermore, the expression profiles of 521 common DEGs were performed in the four libraries using hierarchical clustering (Figure 2C). Gene expression patterns were classified into four main branches according to the clustering analysis. Compared with LC, the upregulated DEGs in LZ were clustered into the first main branches, while the downregulated DEGs in LZ were clustered into the other main branches, suggesting the distinct difference between the mutant and its wild type. Additionally, from 80 to 90 days after flowering, the expression patterns of most DEGs were accumulated in the first branch and the third branch, while the downregulated DEGs were in the second branch and the fourth branch (Figure 2C).

### GO Enrichment and KEGG Enrichment Analyses

**FIGURE 3:**
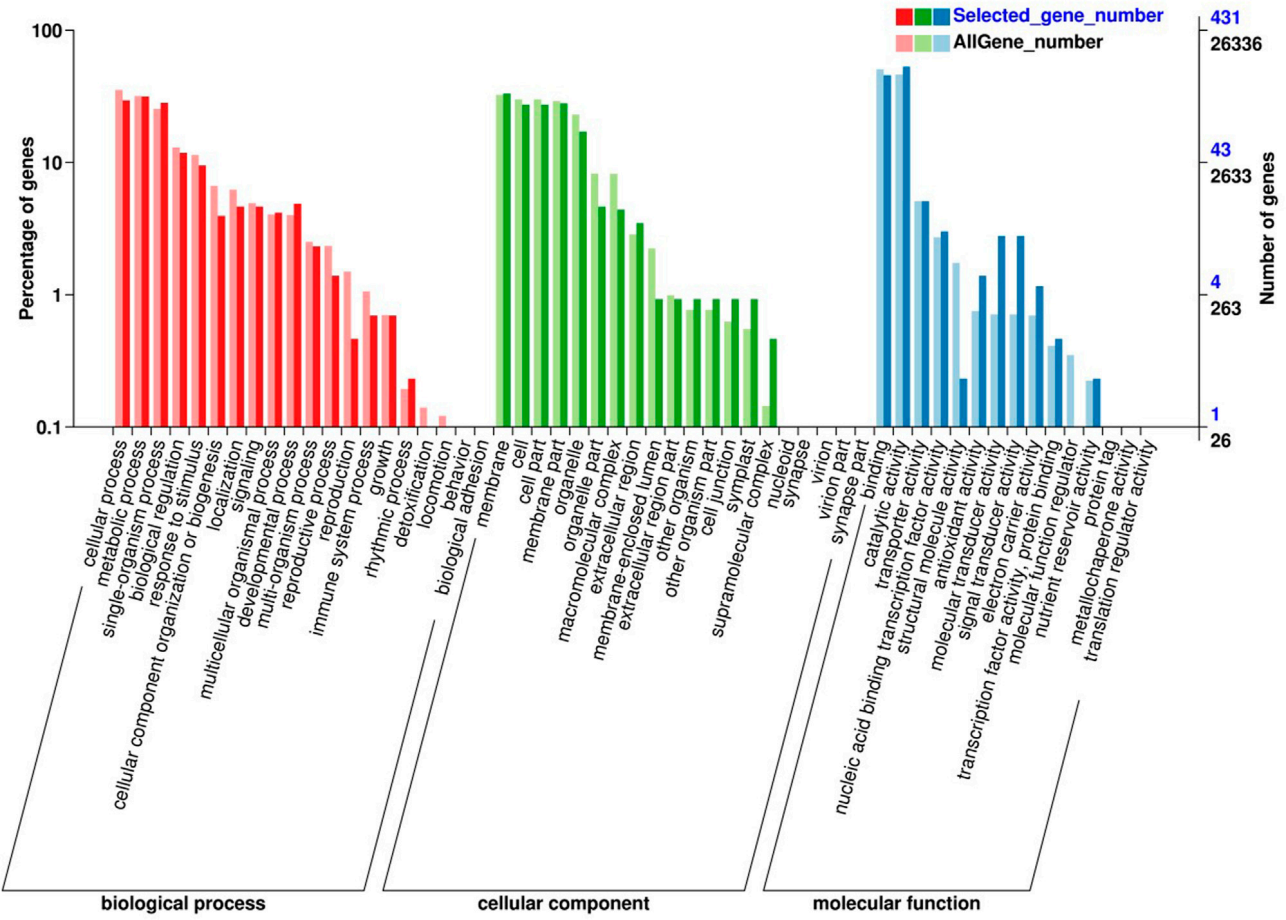
GO enrichment analysis of common DEGs in the early-ripening mutant.

In order to further explore the molecular mechanism of the early-ripening mutant, the common DEGs were examined by GO enrichment and KEGG enrichment analyses. As Figure 3 shows, the common DEGs were annotated into the biological process (BP), cellular component (CC), and molecular function (MF) using GO enrichment. Within the BP category, the DEGs were most enriched in the cellular process, metabolic process, single-organism process, biological regulation, and response to stimulus. In the CC category, the DEGs had significant enrichment in terms of membrane, cell, cell part, membrane part, and organelle. Regarding the MF category, binding, catalytic activity, transporter activity, and nucleic acid binding transcription factor activity were most largely enriched.

**FIGURE 4:**
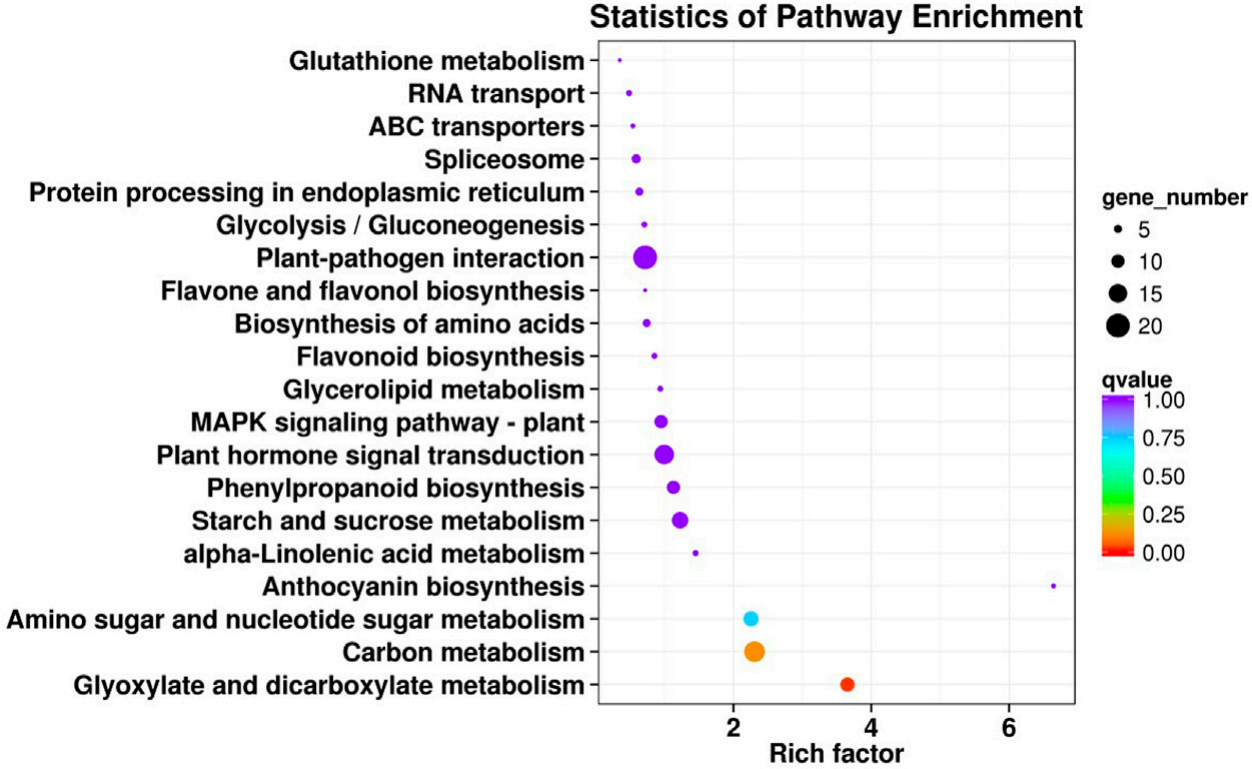
The KEGG enriched scatter plot of common DEGs in the early-ripening mutant LZ. Rich factor refers to the ratio of the DEG number enriched in the pathway to the number of annotated genes. The bigger the Rich factor, the more significant the enrichment is. The q-value is the corrected *p*-value after multiple hypotheses testing, and from red to purple indicates the q-value level.

The KEGG enrichment analysis indicated that 217 DEGs were involved in 101 metabolic pathways. The metabolic pathways were divided into five major metabolic pathway types: cellular process, environmental information processing, genetic information processing, metabolism, and organismal systems; among them, the most enriched pathway was metabolism (Supplementary Figure S1). Moreover, the 20 metabolic pathways with the most significant enrichment of DEGs were shown in Figure 4. The metabolic pathways were mainly related to plant–pathogen interaction (20, 9.22%), carbon metabolism (17, 7.83%), plant hormone signal transduction (16, 7.37%), and starch and sucrose metabolism (13, 5.99%). In addition, a few DEGs were involved in flavonoid metabolism (3, 1.38%) and anthocyanin metabolism (2, 0.92%).

### Identification of Key Candidate Genes Involved in Early Ripening

Fruit ripening is a complex process, attributed to the joint participation of diverse metabolic pathways and genes on these pathways. Based on the KEGG enrichment analysis, the DEG-related plant hormone signal transduction and starch and sucrose metabolism were characterized in Table 2. In the plant hormone signal transduction pathway, 16 common DEGs were involved in the biosynthesis; metabolism; signal transduction; and response of brassinosteroid, jasmonic acid (JA), gibberellin acid (GA), ethylene, and auxin, most of which were downregulated at 80 and 90 days after flowering of the early-ripening mutant LZ, including three genes relating to the JA signaling pathway (jasmonic acid–amino synthetase gene, JAR1; coronatine-insensitive protein 1 gene, COI1; and jasmonate ZIM domain–containing protein gene, JAZ), the GA signal transduction regulatory gene DELLA, and indole-3-acetic acid-amido synthetase GH3.1. Regarding the brassinosteroid signaling pathway, four brassinosteroid insensitive one genes (BRI1s), BRI1-associated receptor kinase 1(BAK1, gene-LOC107423140), and two signal activator cyclin genes (CYCD3s) were downregulated, while the BAK1 gene (LOC107435234) was upregulated at the two fruit development stages of LZ. Meanwhile, three upregulated genes in the salicylic acid (SA) signaling pathway and ethylene signaling were two pathogenesis-related protein 1 genes (PR1s) and ethylene receptor 2 (ETR2), respectively.

For starch and sucrose metabolism, a total of eight upregulated and five downregulated genes were responsive to both 80 and 90 days after flowering (Table 3). Among them, the upregulated common DEGs consisted of four glucanendo-1,3-beta-glucosidase (EGLC), beta-glucosidase (BGLU, gene-LOC107428870), endoglucanase (EGL), beta-fructofuranosidase (INV), and trehalose-phosphate phosphatase (otsB). Conversely, the downregulated common DEGs included glucanendo-1,3-beta-glucosidase (EGLC), beta-glucosidase (BGLU, gene-LOC107412668), isoamylase (ISA), and alpha-amylase (AMY). Since there were no pivotal differentially expressed sucrose and starch synthesis-related enzyme genes, we further analyzed all DEGs between early-ripening mutant and wild-type jujube fruits (Table 3). It was found that five related enzyme genes were specifically differentially expressed at 80 days after the flowering of LZ, including upregulated expression of sucrose synthase gene (SUS, gene-LOC107416188) and three beta-amylase genes (BAM) as well as the SUS gene (gene-LOC107419708) with downregulated expression. It was noted that no gene involved in starch and sucrose metabolism was significantly differentially expressed 90 days after flowering.

**TABLE 3:** DEGs with the annotated functions in plant hormone signal transduction and starch and sucrose metabolism.

Gene ID	Gene name	LC-80d	LC-90d _FPKM	LZ-80d _FPKM	LZ-90d _FPKM	log_2_ (LZ-80d_FPKM/LC-80d_FPKM)	Regulation trend	log_2_ (LZ-90d_FPKM/LC-90d_FPKM)	Regulation trend	Functional annotation
Plant hormone signal transduction										
Brassinosteroid										
gene-LOC107417285	BRI1	9.35	15.97	3.07	6.61	−1.62	down	−1.10	down	Probable inactive receptor kinase RLK902
gene-LOC107418038	BRI1	2.54	2.95	0.41	0.41	−2.40	down	−2.12	down	Squamosa promoter-binding-like protein 13A
gene-LOC107419372	BRI1	3.34	4.08	0.22	1.01	−3.61	down	−1.63	down	Probable inactive receptor kinase At1g48480
gene-LOC107422741	BRI1	9.92	3.55	0.78	0.85	−3.62	down	−1.59	down	MDIS1-interacting receptor-like kinase 2-like
gene-LOC107435234	BAK1	0.45	0.31	10.41	6.46	4.20	up	2.21	up	Leucine-rich repeat protein 1-like
gene-LOC107423140	BAK1	1.81	2.50	0.41	0.13	−1.96	down	−2.54	down	Leaf rust 10 disease-resistance locus receptor-like protein kinase-like 2.4
gene-LOC107426951	CYCD3	21.01	70.86	1.01	5.59	−4.21	down	−3.20	down	Cyclin-D3-1
gene-LOC107419965	CYCD3	0.74	0.97	0.11	0.06	−2.19	down	−1.97	down	Cyclin-D3-1-like
Jasmonic acid										
gene-LOC107424803	JAR1	13.47	13.65	1.02	3.18	−3.64	down	−1.83	down	Uncharacterized protein LOC107424803
gene-LOC107410531	COI1	645.24	349.06	179.04	157.78	−1.87	down	−1.02	down	Histone deacetylase HDT1-like
gene-LOC107417290	JAZ	18.61	37.45	2.02	9.25	−3.01	down	−1.76	down	Protein TIFY 6B-like
Salicylic acid										
gene-LOC107427101	PR1	0.07	0.63	9.61	7.63	6.10	up	2.70	up	Pathogenesis-related protein 1-like
gene-LOC107427103	PR1	0.27	1.17	20.74	18.94	5.65	up	3.14	up	Pathogenesis-related protein 1-like
Gibberellin acid										
gene-LOC107416301	DELLA	4.75	9.27	0.98	2.69	−2.06	down	−1.48	down	DELLA protein GAI-like
Ethylene										
gene-LOC107422981	ETR2	17.45	2.64	53.519	10.69	1.54	up	1.83	up	Ethylene receptor 2
Auxin										
gene-LOC107434645	GH3	39.92	37.35	6.83	9.52	−2.52	down	−1.78	down	Probable indole-3-acetic acid-amido synthetase GH3.1
Starch and sucrose metabolism										
gene-LOC107406007	EGLC	0.04	0.31	2.06	2.60	4.98	up	2.31	up	Glucanendo-1,3-beta-glucosidase 11
gene-LOC107412454	EGLC	2.18	2.14	177.51	78.45	5.60	up	1.79	up	Glucanendo-1,3-beta-glucosidase-like
gene-LOC107412472	EGLC	1.67	1.45	30.39	21.51	4.09	up	3.20	up	Glucanendo-1,3-beta-glucosidase-like
gene-LOC107418979	EGLC	0.15	0.46	23.75	6.83	6.79	up	2.62	up	Glucanendo-1,3-beta-glucosidase
gene-LOC107407180	EGLC	1.66	1.87	0.03	0.03	−4.38	down	−2.31	down	Glucanendo-1,3-beta-glucosidase 2-like
gene-LOC107419097	EGLC	13.08	5.79	1.36	0.84	−3.12	down	−2.01	down	PlasmodesmatA callose-binding protein 3
gene-LOC107428870	BGLU	12.88	17.03	181.25	152.80	3.74	up	2.55	up	Beta-glucosidase 40
gene-LOC107412668	BGLU	8.56	5.05	2.69	1.41	−1.66	down	−1.53	down	Beta-glucosidase 13-like
gene-LOC107410658	EGL	0.39	0.02	2.11	0.57	2.23	up	2.04	up	Endoglucanase 12
gene-LOC107425264	INV	1.15	0.41	27.62	8.60	4.50	up	2.66	up	Beta-fructofuranosidase
gene-LOC107432017	otsB	8.08	9.30	25.63	33.68	1.61	up	1.63	up	Probable trehalose-phosphate phosphatase J
gene-LOC107425983	ISA	3.13	4.68	1.29	1.89	−1.26	down	−1.11	down	Isoamylase 2
gene-LOC107430620	AMY	7.84	11.40	1.41	5.01	−2.44	down	−1.05	down	L-type lectin-domain-containing receptor kinase S.4-like
gene-LOC107416188	SUS	20.79	7.27	107.94	38.38	2.31	up	1.04	normal	Sucrose synthase 5
gene-LOC107419708	SUS	2.06	0.49	0.23	0.10	−2.87	down	−1.20	normal	Sucrose synthase 7-like isoform X1
gene-LOC107406235	BAM	3.39	11.40	12.52	11.27	1.78	up	0.05	normal	Beta-amylase 7 isoform X1
gene-LOC107422617	BAM	117.08	79.69	323.64	88.31	1.43	up	0.21	normal	Beta-amylase 1
gene-LOC107430415	BAM	7.44	9.38	39.46	4.81	2.35	up	−0.83	normal	Beta-amylase 3

**FIGURE 5:**
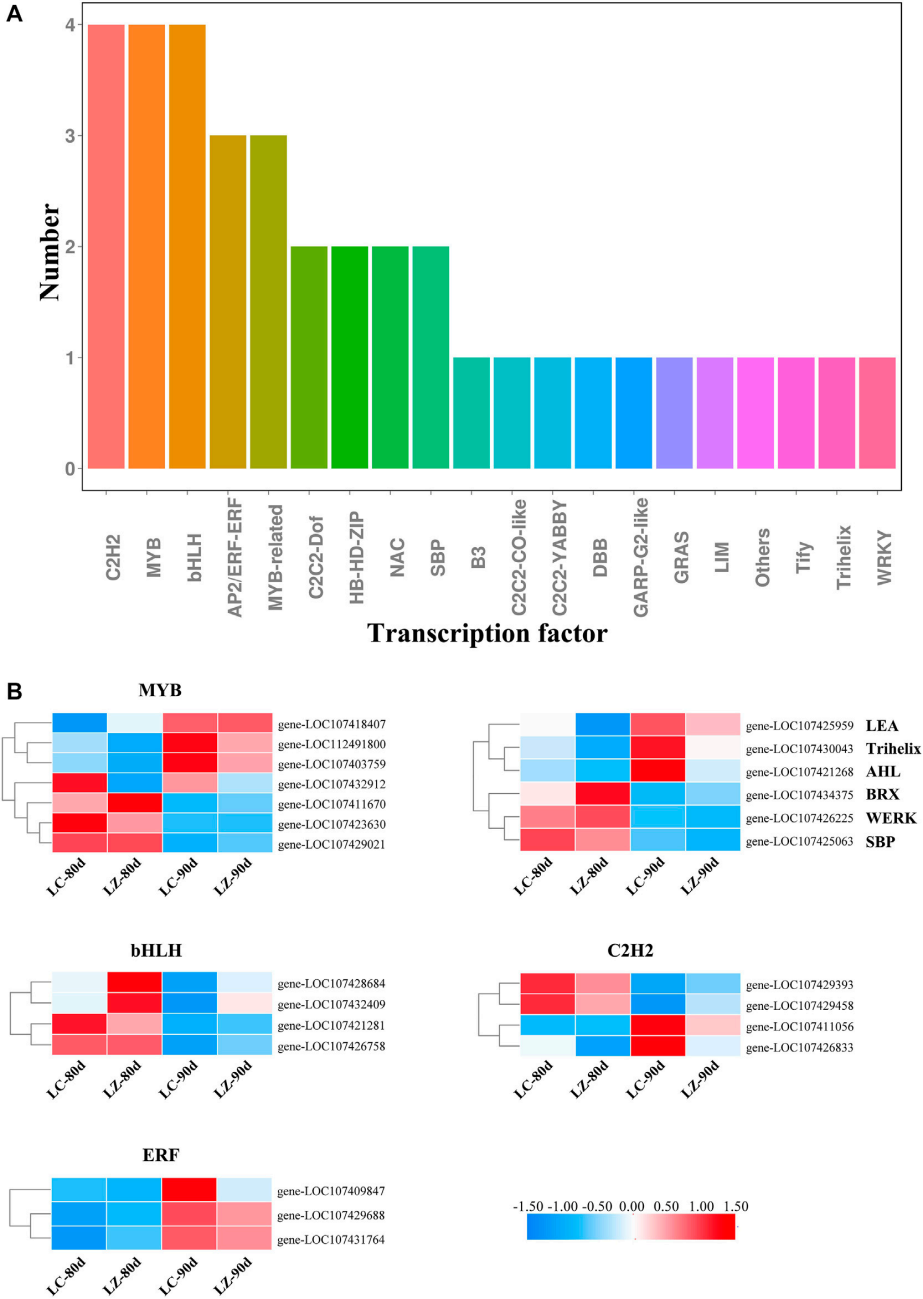
The classification of transcription factor family members and their differential expressions during the late fruit-ripening stage. **(A)** Classification of transcription factor family. **(B)** Differential expressions of main transcription factor family members.

Transcription factors play a very important role in fruit growth and development. According to the classification of transcription factor families, 24 differentially expressed transcription factor genes were abundant in 20 families during the later fruit maturity stage. From Figure 5A, the members of C2H2, MYB, and basic helix–loop–helix (bHLH) families were the most with four, respectively, along with three AP2/ERF-ERF genes and three MYB-related genes. C2C2-Dof, HB-HD-ZIP, NAC, and SBP families contained two members, respectively. Furthermore, as shown in Figure 5B, the transcription expressions of three MYBs (gene-LOC107418407, gene-LOC112491800, and gene-LOC107403759), three ERFs (gene-LOC107409847, gene-LOC107429688, and gene-LOC107431764), two C2H2s (gene-LOC107411056 and gene-LOC107426833), one Trihelix (gene-LOC107430043), one AHL (gene-LOC107421268), and one LEA (gene-LOC107425959) were significantly accumulated in LZ. Conversely, 12 downregulated transcription factors in ‘LZ’ were three MYBs (gene-LOC107411670, gene-LOC107423630, and gene-LOC107429021), four bHLHs (gene-LOC107421281, gene-LOC107426758, gene-LOC107428684, and gene-LOC107432409), two C2H2s (gene-LOC107429393 and gene-LOC107429458), WRKY (gene-LOC107426225), SBP (gene-LOC107425063), and BRX (gene-LOC107434375). Collectively, 23 transcription factors appeared to the distinct difference between LZ and LC. However, only one MYB (gene-LOC107432912) expression trend was similar in the two jujube cultivars, showing the downregulated expression as the fruit maturity.

### DEGs Validation Using RT-PCR Method

**FIGURE 6:**
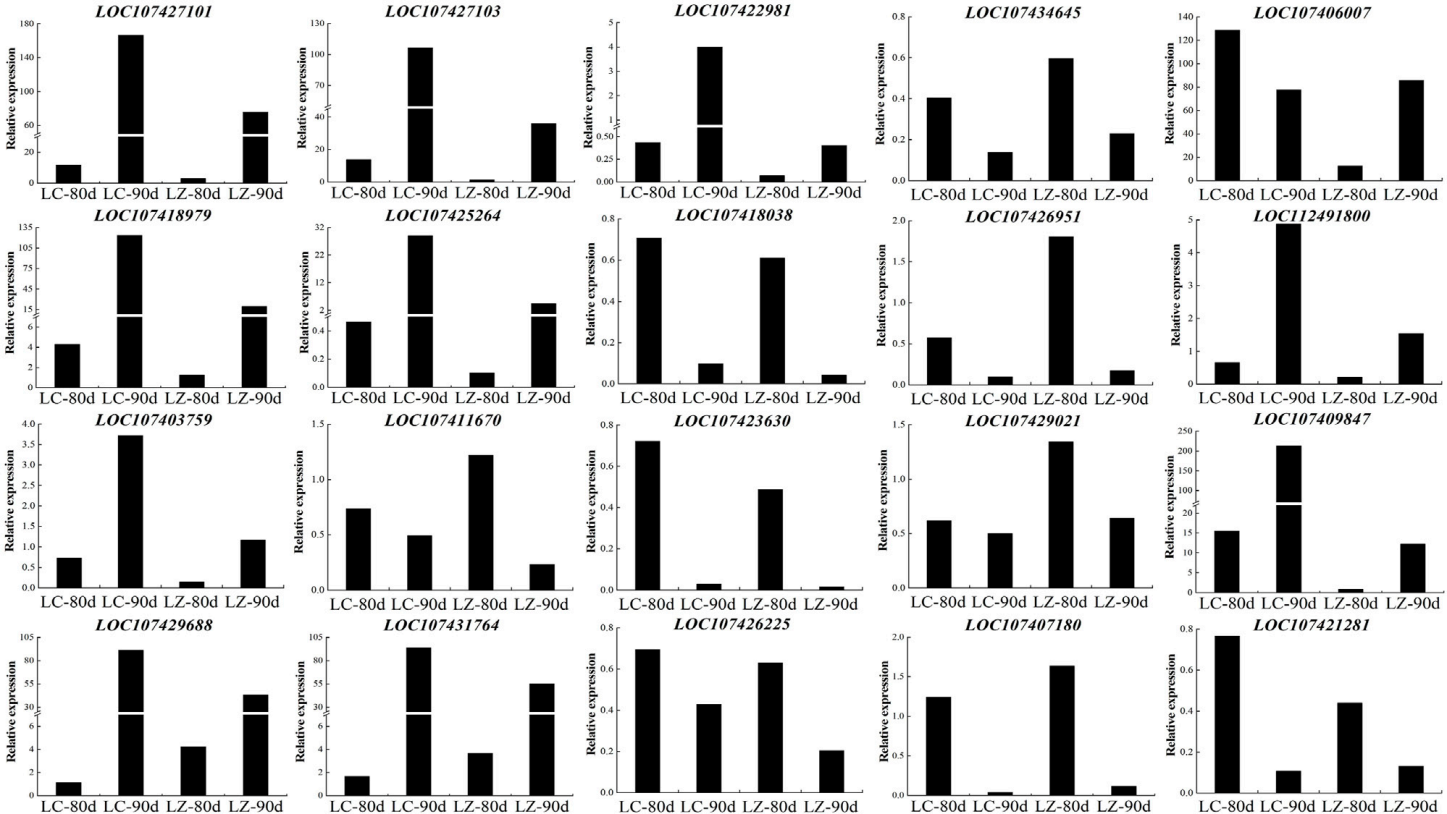
Expression profiles of twenty randomly selected DEGs from LZ and its mutant LC jujube cultivars using real-time quantitative PCR.

To assess the repeatability of the RNA-Seq sequencing data, the randomly selected 20 genes detected the gene expression level at 80 and 90 days after flowering between LC and LZ (Figure 6). The results showed that the expression patterns of the 20 genes were consistent with the respective RNA-Seq data, which confirmed that our RNA-seq expression data and subsequent interpretations were reliable.

## Discussion

Fruit ripening is a complex process. In general, the skin color turns red during the jujube fruit’s development, which is considered one of the ripening traits (Shi et al., 2018). Combined with our survey from 2016 to 2021, the skin color of LZ showed red color and matured earlier than LC (Figure 1A), so LZ was considered the early-ripening mutant. The higher SSC and lower titratable acid content in LZ fruit might be signals to induce ABA synthesis, which was consistent with the report by Wheeler et al. (2009). Meanwhile, we found higher ABA content during the ripening process of LZ, compared with LC. It was deduced that ABA was a promoter of jujube ripening. The ripening of the Chinese jujube is non-climacteric (Zhang et al., 2018). The ACC content reached the peak before the maturity stage and was higher in LC than in LZ (Figure 2B), though the ethylene receptor 2 gene (LOC107422981) was upregulated at 80 and 90 days after flowering in LC, which suggested that ethylene level was not pivotal for the non-climacteric fruit ripening (Wheeler et al., 2009; Zhang et al., 2018). It was noted that ABA metabolism played a core accelerating role in fruit ripening rather than ethylene.

In the present study, 521 common genes were identified from 80 to 90 days after the flowering of LZ and LC fruits. GO analysis revealed that the DEGs were involved in cellular process, metabolic process, single-organism process, biological regulation, and so on. Combined with KEEG enrichment analysis, the DEGs were most significantly enriched in 20 metabolic pathways, particularly, carbon metabolism and plant hormone signal transduction.

The accumulation of glucose and fructose was integral to grapevine ripening (Castellarin et al., 2011), and glucose and fructose were at high concentrations in LC jujube fruits, which were determined using the HPLC–ELSD method (Guo et al., 2015.). Our finding indicated that 18 DEGs in starch and sucrose metabolism were identified, including 12 upregulated and six downregulated at 80 days after the flowering of LZ (Table 3); furthermore, eight among the 12 upregulated genes kept being expressed at a higher level at 90 days after the flowering of LZ. Collectively, the eight genes (LOC107406007, LOC107412454, LOC107412472, LOC107418979, LOC107428870, LOC107410658, LOC107425264, and LOC107432017) related to glucose and fructose had a closer relationship with jujube early ripening.

Brassinosteroids (BRs) are a group of naturally produced steroid phytohormones regulating plant growth and development, and stress responses. BR deficiency delayed flowering time and leaf senescent (Gou et al., 2012; Zhu et al., 2015). The overexpression of the tomato BRI1 (BR-insensitive 1) gene promoted fruit ripening and ethylene production during fruit ripening of the transgenic plants (Nie et al., 2017). However, regarding plant hormone signal transduction, seven among eight genes from brassinosteroid expressed a downtrend during fruit maturity of LZ, compared with LC, including four BRI1 genes (Table 3), which indicated that the seven genes related to brassinosteroid signal transduction were the key candidate genes leading to the maturity period of LC later than LZ. Furthermore, keeping in view that BRI1 cannot initiate BR signaling without BAK1 and its homologues (Nam and Li. 2002; Gou et al., 2012), BAK1 (LOC107435234) might play a more significant role by interacting with BRI1 genes in jujube fruit ripening.

Transcription factors can activate or repress the transcription of downstream target genes by binding specifically to the promoters of the target genes, regulating a wide range of biological processes as well as the plant response to abiotic and biotic stresses (Singh et al., 2002; Ramamoorthy et al., 2008). 80 and 90 days after flowering, 24 differentially expressed transcription factor genes were characterized in our current study. Qing et al. (2019) identified 171 MYB superfamily genes in the jujube, and the MYB-related subfamily participated in different regulatory mechanisms during jujube fruit development. Based on RNA-Seq profiling between the Hanfu apple and its mutant, 8 DEGs among 26 transcription factors were speculated to be implicated in the hormonal regulation and anthocyanin biosynthesis during apple fruit ripening (Nawaz et al., 2021). Also, MYB and bHLH genes more commonly resulted in color differentiation in plants (Zong et al., 2019). On behalf of a large transcription factor family, such as MYB and bHLH, it was difficult to elucidate a clear biological function for a certain phenotype using a single gene. Thus, during the later stage of jujube fruit maturity, three upregulation MYB genes (LOC107418407, LOC112491800, and LOC107403759), three downregulation MYB genes (LOC107411670, LOC107423630, and LOC107429021), and four bHLH genes (LOC107421281, LOC107426758, LOC107428684, and LOC107432409) may be involved in modulating anthocyanin biosynthesis during jujube fruit ripening together. ERFs as important plant-specific transcription factors, located at the end of the ethylene signaling pathway, mediated ethylene-regulated gene expression (Liu et al., 2016). Zhang and Li (2018) identified 119 AP2/ERF genes from the Chinese jujube genome, including 57 ERFs. Moreover, ZjERF54 positively participated in jujube fruit ripening, while ZjERF25 and ZjERF36 were ripening repressors. We found that three ERF genes (gene-LOC107409847, gene-LOC107429688, and gene-LOC107431764) had the same expression profiles as the ACC content change during the late fruit maturity stage; furthermore, 80 days after flowering, the genes exhibited higher transcript abundance in LZ than in LC, suggesting that the three ERFs were crucial candidate genes for regulating ethylene biosynthesis. C2H2 transcription factors are a large family in eukaryotes involved in the ripening of economically important fruits. However, *MaC2H2-1* and *MaC2H2-2* genes may negatively regulate ethylene production during banana fruit ripening (Han et al., 2016). However, the jujube fruit is a non-climacteric fruit, with different expression trends of four C2H2 genes in the current regulated ethylene production; thus, how C2H2 genes are involved in jujube fruit ripening via regulating ethylene production still needs further study.

## Conclusion

The present study is the first to explore the early ripening of jujubes using transcriptome analysis. A total of 5,376 and 762 DEGs were identified at 80 and 90 days after the flowering of the jujube fruit from ‘Lingwu Changzao’ and its early-ripening mutant ‘Lingzhou No.1’, respectively. Furthermore, among 521 common DEGs, the eight upregulated genes related to glucose and fructose, the seven downregulated genes related to brassinosteroid signal transduction, and a range of transcription factor genes (MYB, bHLH, and ERF) likely accelerated the fruit ripening of jujubes together. Additionally, jujube is a non-climacteric fruit where ripening is under the control of ABA rather than ethylene. These findings contributed to explanation of the mechanism of early ripening for non-climacteric fruit crops.

## Data Availability

The datasets presented in this study can be found in online repositories. The names of the repository/repositories and accession number(s) can be found at: https://www.ncbi.nlm.nih.gov/search/all/?term=PRJNA811791.
