# Supplementary material for: Transcriptome Profiling and Identification of the Candidate Genes Involved in Early Ripening in Ziziphus Jujuba
Source: Front Genet. 2022 Jun 14;13:863746. doi: 10.3389/fgene.2022.863746 (PMC9237510; doi:10.3389/fgene.2022.863746)
Supplement: Supplementary file 1 [file Table1.DOCX]

Table S1 Primers for Real-time PCR used in the current study

| **Gene ID** | **Froward primer (5' to 3')** | **Reverse primer (5' to 3')** |
| --- | --- | --- |
| *18S* (LOC112490218) | CAACCATAAACGATGCCGA | AGCCTTGCGACCATACTCC |
| LOC107427101 | TGACTACAACTCCAACTCCT | TCCATTGTCGCACTTAACC |
| LOC107427103 | ACAACTCCAACTCCTGTG | CCTCCATTGTCGCACTTA |
| LOC107422981 | GATGTCGGCAGATTGGAAT | GTGCCTTCACACAACTTTG |
| LOC107434645 | AGTACATTCCAACGCTTGA | GCACATTGGTTTGAGGTTT |
| LOC107406007 | GAGGTTGGAAGTCTATCTGTT | CCACATTGTAAGCCATAGTTC |
| LOC107418979 | ATTCGTCATGTTAGAGGAGGA | CCCAAAGTGTTTCTCCAGTT |
| LOC107425264 | GTCACTAAGAAGCCTGATTGAT | CGTTCAGTAGCCATTATTGGA |
| LOC107418038 | TCACTGTCCAGGAATGTT | GTGAAAGGGAAGTGTTTGA |
| LOC107426951 | TCCTTTCAACACTCCAATGG | TTCAATCCAAGCCTCCTTATG |
| LOC112491800 | GCTCAAATGTCAACAATGGT | TGTTCAACATGGTAATCTCCT |
| LOC107403759 | CAAATGTCAACAATGGTTCCTC | TGTTCAACATGGTAATCTCCTG |
| LOC107411670 | TCACCATCATCAGAACCA | AAGCTACAGCAAGTCTCT |
| LOC107423630 | CATCAACATCCTCTGAATGGC | GCTGAAGGTATTCCTCGTACA |
| LOC107429021 | GAAACCTGGATTGGATAACATCT | TCCTTGGTCCAGACATATACAA |
| LOC107409847 | AGCGTTCCTTTCTTTAATCCC | GTTGTTGTCCTCCATTGAAGT |
| LOC107429688 | CTTCCACATCTTCCTCTTCA | GCGATCATCATCATCATCATC |
| LOC107431764 | CTTCCACATCTTCCTCTTCA | GCGATCATCATCATCATCATC |
| LOC107426225 | GGCACCCCATGTACTTAAAG | GCTAAGGCAGCAGTGAAAT |
| LOC107421281 | TTGAAGGCTGTAGTGGAGAA | TTCATCCTCACCTTCGCTAA |
| LOC107407180 | TCAATGAATTCTACGAGTGCTG | GCACAAAAGAATCCCAATAACC |
